# VIP in HIV Diarrhea: Finding Links for the “Slim Disease”

**DOI:** 10.3389/fphys.2015.00402

**Published:** 2015-12-23

**Authors:** Arun Chaudhury

**Affiliations:** GIM Foundation and Arkansas Department of HealthLittle Rock, AR, USA

**Keywords:** AIDS, chronic diarrhea, vaccine, neuropeptide, Treg, macrophage

Diarrhea, in the context of human immunodeficiency virus (HIV) infection, is an enormously important clinical issue (Smith et al., 1988). Diarrhea in patients with AIDS is extremely problematic for both patient and clinician. More than 50% of AIDS patients suffer from diarrhea, the cause of which is unexplained in most of the cases. The causes of diarrhea in HIV infection are multifactorial. Chiefly, these may be caused by the primary retroviral infection *per se*, several opportunistic infections, alteration of gut microbiome, immune reconstitution, water sanitation or as a complication of highly active anti-retroviral therapy (HAART) like didanosine or ritonavir, and may also result from induced pancreatic insufficiency (Haas et al., 2011; Saha et al., 2011; Murray and Rubio-Tapia, 2012; Vyas et al., 2012; Agholi et al., 2013; Martin et al., 2013; Clay and Crutchley, 2014; Dye, 2014; Dikman et al., 2015; Green et al., 2015; Rubaihayo et al., 2015; Yates et al., 2015). The diarrhea in primary HIV infection is primarily secretory in nature (Juckett and Trivedi, 2011; MacArthur and DuPont, 2012). Some preliminary studies have suggested the role of viral proteins in causation and aggravation of diarrhea in HIV infected patients. For example, retroviral tat proteins have been demonstrated to enhance myenteric neuronal excitability by altering different sodium channels (Ngwainmbi et al., 2014). It is obvious that in HIV infection, a viral specific protein that affects genomic transcription would affect diverse cellular mechanism (s). This opinion piece advances a concept of how HIV may gain entry into the gastrointestinal mucosa using an upstream neuropeptide mechanism, providing a perspective of an incipient mechanism involved in mucosal disease caused by HIV and potential druggable targets for HIV induced diarrhea.

Namely, the hypothesis is based on two key reported observations: (i) the molecular overlap of a pentapeptide belonging to gp120, the key viral envelope protein that helps in cellular docking and a segment of vasoactive intestinal polypeptide (VIP) (Pert et al., 1988). HIV have been variously reported to bind to the VIP receptors, VPAC1 and VPAC2, and produce differential effects (see below). (ii) The earliest observations from HIV infected patients of the elevation of serum VIP during primary HIV illness and diarrhea (Manfredi et al., 1993, 1994). Potentially, how primary mucosal HIV infection causes elevation of serum VIP in not known. Here a rationale is presented that has striking similarity to a mechanism similar to *Clostridium difficile* diarrhea, one which involves disruption of cytoskeletome of mucosal epitheliocyte (Farrell and LaMont, 2000; Kumar et al., 2014). The tat proteins, through transcriptional activation, may hijack and alter the cytoskeletal machinery, thus resulting in leakage of cellular peptides like VIP (Vitale et al., 2013). This may also occur from submucosal neurons which are rich in VIP (Vitale et al., 2013). It is well known that excessive VIP in the mucosal extracellular space may cause secretory diarrhea (Krejs et al., 1980; Chambers et al., 2005), much like that seen in VIPoma (Krejs et al., 1977). There are only scant reports which have examined the electrolyte composition of diarrheal stool in HIV diarrhea or examined mucosal flux of electrolytes by Ussing's chamber after retroviral mucosal loading, but is generally agreed that it is of the secretory type (Schiller et al., 1994; Stockmann et al., 2000; Nwachukwu and Okebe, 2008).

The initial rise in VIP secretion may be related to a beneficial microenvironment of immune resistance. VIP have been reported to reduce viral production in HIV-1-infected human primary macrophages (Souza et al., 2014). The exact mechanisms of transfer of HIV-1 into gut mucosal and nerve terminals is not precisely known. Importantly, HIV-1 does not replicate in neurons and its productive infection in other cell types leads to the release of neurotoxic viral proteins, such as Tat, Nef, Vpr, and gp120 (Souza et al., 2014). These peptides may cause neuronal apoptosis. These neuroplastic aspects affecting the enteric nervous system (ENS) merits further studies. VIP exerts anti-inflammatory activity by inhibiting the activity or production of the interleukins IL-12, IL-6, IL-1β, tumor necrosis factor (TNF-α) and macrophage migration inhibitory factor, and stimulate the production of anti-inflammatory factors like IL-4 and IL-10. VIP and PACAP also participate in Th2 cell differentiation and inhibit synthesis of the Th1 cytokines IFN-γ and IL-2 (Souza et al., 2014). Additionally, VIP induces tolerance development in dendritic cells, induces the differentiation of T regulatory cells (T_regs_) and reduces TLR2 and TLR4 expression on CD4+T cells (Chorny et al., 2006; Delgado and Ganea, 2013). It is known that viral antigen-induced cell death (AICD) is mediated by the T cell receptor (TCR) and involves Fas/Fas ligand (FasL) interactions. By affecting the expression of FasL. VIP may play significant role in the inhibition of FasL-mediated T cell cytotoxicity (Delgado and Ganea, 2000) and potentially alter the course of primary mucosal HIV infection. An important area of investigations would be how the intraepithelial lymphocytes and dendritic cells fail to mount adequate antiviral response after the initial infections and the role of CD25-FoxP+ve T_reg_ cells (Wang et al., 2015).

The small homology (five amino acids) between VIP peptide sequences and the variable region V2 of HIV-1 gp120 (Sacerdote et al., 1987) suggest that VIP and their receptors might potentially participate in the pathogenesis of AIDS. These initial suggestive hypothesis came during exploration of possible mechanisms of neurocognitive complications that arose in the HIV infected populations (before the era of HAART) (Ngwainmbi et al., 2014). It has been proposed that VIP could block HIV-1 entry into target cells through direct interaction with the CD4 molecule (Souza et al., 2014). VIP also could act as a blocking peptide by binding to gp120, and prevent HIV binding to target cells. Additionally, these peptides may modulate transfer of HIV from macrophages/dendritic cells to mucosal epitheliocyte and submucosal neurons. These aspects merits systematic testing.

VIP receptor activation by HIV-1 produces opposing effects. It has been reported that productive HIV-1 infection can be facilitated by the sole activation of VPAC1, one of the major receptor classes for VIP (Bokaei et al., 2007). On the other hand, viral production is reduced in HIV-1-infected cells either following the engagement of VPAC2, the other major receptor for VIP (Branch et al., 2002). However, when cells are treated with VIP, with resultant engagement of both VPAC1 and VPAC2, HIV-1 production is reduced in the target cells (Temerozo et al., 2013). An initial pilot study could not detect the effect of VIP receptors (Nguyen, 1988). The precise mechanism for this disconcordant observation is not known but may have resulted from lack of examination of the multitude of splice variants of VIP receptors (Bokaei et al., 2006) and differential selectivity of peptide fragments and full-length VIP sequence on binding affinity to its receptors (Rorstad et al., 1990).

If mucosal ulcer occurs with HIV infections and viral entry is not well known. In other organs, HIV is known to cause epitheliocyte damage. For example, HIV associated nephropathy (HIVAN) is associated with collapsing glomerulitis (Rosenberg et al., 2015). This epithelial damage is known to be associated with alteration of transcription factors like snail (Kumar et al., 2011). Snail regulates the genomic expression of different kinds of myosin by acting on its promoter (Chaudhury et al., 2014). Whether HIV alters myosin promoter expression and functions after retroviral genome integration merits further investigation.

Additional hypotheses have been posited about HIV mucosal disease. Preliminary suggestions have been made about morphine based mechanisms of HIV mucosal illness, based on empiric observations of worsening HIV primary disease in opioid abusers (Samikkannu et al., 2015; Sindberg et al., 2015; Weisberg et al., 2015). This correlation may not be necessarily be mechanistic, as morphine based mechanism in general inhibit oro-aboral transit of luminal contents and anti-diarrheal in its summative pharmacologic manifestations. Furthermore, apart from opioid abuse, injection drug use may produce a number of confounding situations in HIV infected people including hepatitis C virus (HCV) coinfections. A common route of HIV entry, apart from being in circulation, are sexual practices like anal penetration exercised by preferences like men having sex with men (MSM) (Wahome et al., 2013). Whether such acts cause mucosal entry and acts as a nidus for HIV entry and how VIP contributes to these process merits further exploration. HIV infected populations are exposed to the risk of multiple sexually-transmitted infections (STI), including syphilis (Jia et al., 2015; Taylor et al., 2015), and which is prevalent in epidemic proportions across the globe. Syphilis may cause Crohn's like colitis-type proctitis (Davis and Goldstone, 2009; Lamb et al., 2013), especially with prevalent sexual practices, and these mucosal lesions may also favor HIV dissemination through the inflamed mucosa.

The first-in-class agent crofelemer has been recently FDA (US Food and Drug Administration) approved to manage HIV and HAART- induced non-infectious diarrhea (Crutchley et al., 2010). Crofelemer derived from extract of *Croton lechleri* (dragon's blood, *Sangre de Drago/Sangre de Grado*) blocks the chloride channels CFTR and TMEM16A. This is an end point mechanism for inhibiting chloride secretion-induced water drag and resulting secretory diarrhea. VIP in the extracellular space may be related to creating an antiviral environment, but at the cost of inducing secretory diarrhea, which upon chronic persistence may often be fatal and contribute to marked cachexia. Some recent hypotheses have suggested the plausible role of ENS-stimulated VIP release and its protective effects on intestinal epithelial barrier (Ben-Horin and Chowers, 2008). HIV has also been proposed to cause non-malabsorptive diarrhea by affecting leak flux mechanism in a small series of duodenal biopsies (Stockmann et al., 1998). The effect of HIV infection on barrier function is an important area of future investigation. If the VIP receptor mechanisms for HIV viral entry into the gastrointestinal mucosa are further validated, blocking the pentapeptide by heteroclitic peptide or a blocking antibody could become a simple but potent vaccine strategy to prevent HIV diarrhea and control persistence and spread of the retrovirus.

In summary, HIV associated diarrhea remains a major cause of morbidity and mortality even now, with presentations comparable to the earliest report of “slim disease”, the cachectic manifestation, from rural Uganda (Serwadda et al., 1985). Public health measures and reductionistic bench side approaches are needed for appropriate rationale management of this global pandemic of HIV- associated diarrhea.

## Conflict of interest statement

The author declares that the research was conducted in the absence of any commercial or financial relationships that could be construed as a potential conflict of interest.
